# Exercise ameliorates stanozolol and cannabis co-abuse testicular damage in rats evidenced by biochemical and histological regulation of SIRT1 and STS

**DOI:** 10.1038/s41598-025-31785-6

**Published:** 2025-12-27

**Authors:** Shaimaa ElShebiney, Noha A. Mowaad, Rania Elgohary, Marwa E. Shabana, Rasha S. El-Mahdy, Asmaa M. Elfiky

**Affiliations:** 1 Narcotics, Ergogenics and Poisons Department, Medical Research and Clinical Studies Institute, National Research Centre, Cairo, Egypt; 2 Department of Pathology, Medical Research and Clinical Studies Institute, National Research Centre, Cairo, Egypt; 3 Children with Special Needs Department, Medical Research and Clinical Studies Institute, National Research Centre, Cairo, Egypt; 4 Environmental and Occupational Medicine Department, Environment and Climate Change Research Institute, National Research Centre, Cairo, Egypt; 5 33 El-Buhouth st., Cairo, P.O.12622, Dokki Egypt

**Keywords:** Stanozolol, Cannabis, MPO, Testicular (NAG), iNOS, Caspase-3, SIRT1, STS, Biochemistry, Cell biology, Endocrinology, Physiology

## Abstract

Polydrug use among teenagers is widespread and emergent either among athletes or non-athletes. It is reported that stanzolol (Stanz) is commonly abused with cannabis (Cann), this combination probably affects the testicular functions negatively. Aim The present study aimed to evaluate the toxic effects of Stanz and or Cann on reproductive hormones and testicular enzymes. Male Wistar rats were administered Stanz (5 mg/kg, s.c., once per week) and Cann (20 mg/kg, i.p., daily) either alone or in combination for two months, in exercise or sedentary conditions. Swimming exercise protocol was applied. Administration of both Stanz and Cann induced testicular damage, as evidenced by altered hormones, oxidative stress, and testicular enzymes. The testis tissue was significantly injured by the combined administration. In serum, levels of free testosterone, follicular stimulating hormone (FSH), lutenizing hormone (LH) were markedly reduced, while sorbitol dehydrogenase level increased. Moreover, tissue malondialdehyde (MDA) was significantly increased, glutathione (GSH) content decreased, testicular N-acetyl-β-glucosaminidase (NAG) and Myeloperoxidase (MPO) were increased. SIRT1 and STS mRNA expression were downregulated. Besides, distinct histopathological changes were detected in testis of Stanz and Cann injected rats. Nevertheless, Stanz, Cann or combined treatment showed a considerable up-regulation of immunoexpression of inducible nitric oxide synthase (iNOS) and caspase-3 in testes tissue. Oxidative stress and inflammation played a significant role in the observed pathological changes. Training was partially ameliorating for the observed effects. Use of the drugs in sedentary rats had more detrimental effects on testes. Although exercise could palliate the damage partially, it was not fully protective.

## Introduction

Anabolic androgen steroids (AAS) such as stanozolol (Stanz) are used as therapeutic aids for multiple medical conditions, whereas athletes or even sedentary people use AAS to perfect their physical appearance and muscular performance^1^. 4 to 6% of exercising men abuse AAS^2,3^. The general practice is in cycles of supraphysiological doses which lead to fluctuating sexual dysfunction and depressed mood with emotional instability. These cycles predispose to dependency that around 6% to 20% of abusers escapes withdrawal symptoms by using another recreational drug such as cannabis^4^.

Androgen receptors (AR), members of the nuclear receptor superfamily are found in every tissue with varying affinities to AAS. Their anabolic effects are linked to glucocorticoid antagonistic effects, growth hormone (GH) stimulation, insulin-like growth factor 1 (IGF-1) production and psychoactive effects on the brain are not avoided^5^. Depending on the dosing regimen, they can have a number of sides effects, such as cardiovascular diseases, sudden cardiac death, acute hepatitis, jaundice, hypertension^6^, male infertility, and behavioral issues including excessive sexual stimulation^7^. Stanz, a synthetic testosterone 17-alkylated derivative, is most commonly abused among AAS^8^.

Cannabis has long been linked to sports and was included in the Olympic Victors Dark Ointment, which was used as a pain reliever at the first Olympics^9^. Zeiger et al.^10^ revealed that over 60% of the athletes in the study sample had used cannabis at least once in their lives, and over 20% continued to use it for both pain management and recreational purposes endorsing the perception about it as a powerful pain-inhibitor^11^. Cannabis has over 60 active phytocannabinoids, the most significant of which are cannabidiol (CBD) and tetrahydrocannabinol (THC). THC is interrelated to the psychological effects however, cannabis-related risks are often underestimated^12^.

Athletes and regular exercisers gradually get used to their training schedule and become less vulnerable to oxidative damage, nonetheless lengthy and intensive workouts can lead to deleterious consequences of exercise-induced free radical flow^13^. Though exercise is advantageous to almost every physiological system, including the reproductive system, the kind, frequency, and intensity of exercise affect its health benefits^14^. There is a common belief among youth that the use of these drugs would not be risky as long as supplemented with an intense physical exercise. Smit and de Rond (2023)^15^ concluded that entering the path of doping cycles induce a continued use pattern despite any health hazard.

The hypothalamic–pituitary–gonadal (HPG) axis is the central regulatory pathway of male reproductive function, controlling testosterone synthesis, spermatogenesis, and overall testicular activity. Disruption of this axis can result in profound reproductive impairment. Anabolic–androgenic steroids (AAS) such as stanozolol (STZ) exert negative feedback on the HPG axis by suppressing the hypothalamic release of gonadotropin-releasing hormone (GnRH), which consequently reduces pituitary secretion of luteinizing hormone (LH) and follicle-stimulating hormone (FSH). This suppression leads to decreased intratesticular testosterone levels and impaired spermatogenesis^2^. Similarly, cannabinoids (CB), the active components of cannabis, interact with the endocannabinoid system, which is closely linked to the HPG axis. Activation of cannabinoid receptors (particularly CB1 receptors) in the hypothalamus and testis can inhibit GnRH release and reduce LH and FSH secretion, thereby impairing steroidogenesis and sperm production. Chronic exposure to either substance—and particularly their co-abuse—may therefore synergistically disrupt endocrine signaling, promote oxidative stress, and lead to testicular atrophy and dysfunction^16^.

Considering the capacity to modulate physical performance and the risk of negative health effects, including serious effects on testicular tissue and reproductive function, the use of muscle-boosting AAS in conjunction with intentional or recreational misuse of cannabis in sport should be taken into consideration. This study was designed to close the gap in the literature by examining the combined toxic effects of stanozolol and cannabis in male albino rats, Therefore, experimental animal models—particularly male albino rats—are widely employed to simulate and investigate the underlying biochemical, histological, and molecular alterations associated with such drug exposure. This approach allows for controlled assessment of testicular toxicity, oxidative stress responses, and gene expression changes that mirror those observed or hypothesized in human athletes, This is the primary goal of this study. on athletes.

## Materials and methods

### Drugs and chemicals

Injectable stanozolol solution was procured from ZPHC (USA). Cannabis resin was attained from the Azbakeyya Criminalistics Department, by licence from Ministry of Justice (seizure number 809/2019, Cairo, Egypt). Chloroform was used to extract the resin, then dried and ground into a powder. Saline was used to dissolve 10 mg/ml of the dried resin powder, which was then intraperitoneally (i.p.) injected^17^.

### Experimental design

A total of 48 male Wistar albino rats, aged 2–3 months and weighing approximately 200–250 g, were sourced from the breeding colony at the National Research Centre, Cairo, Egypt. The rats were housed in polypropylene cages (4 per cage) and maintained on a 12-hour light-dark cycle in controlled environment (20–23 °C). They were provided with ad libitum access to a commercial high-protein rat diet and tap water. Prior to the start of any experimental procedures, a one-week acclimatization period was allowed. The study received ethical approval from the National Research Centre Medical Research Ethics Committee (Approval No. CU-II-F-10–18) and was conducted in accordance with the National Institutes of Health’s Guide for the Care and Use of Laboratory Animals (Publication No. 85 − 23, revised 1985) in line with ARRIVE guidelines. Animal welfare was given first attention from the beginning of the investigation until its conclusion. Pain and discomfort were prevented, reduced, or eliminated whenever feasible by a number of proactive and caring techniques. As the study went on, each animal was intensively observed, first once a day and later twice a day. To ensure professional monitoring, these welfare assessments were carried out by a qualified laboratory animal scientist^18,19^.

**Figure:**
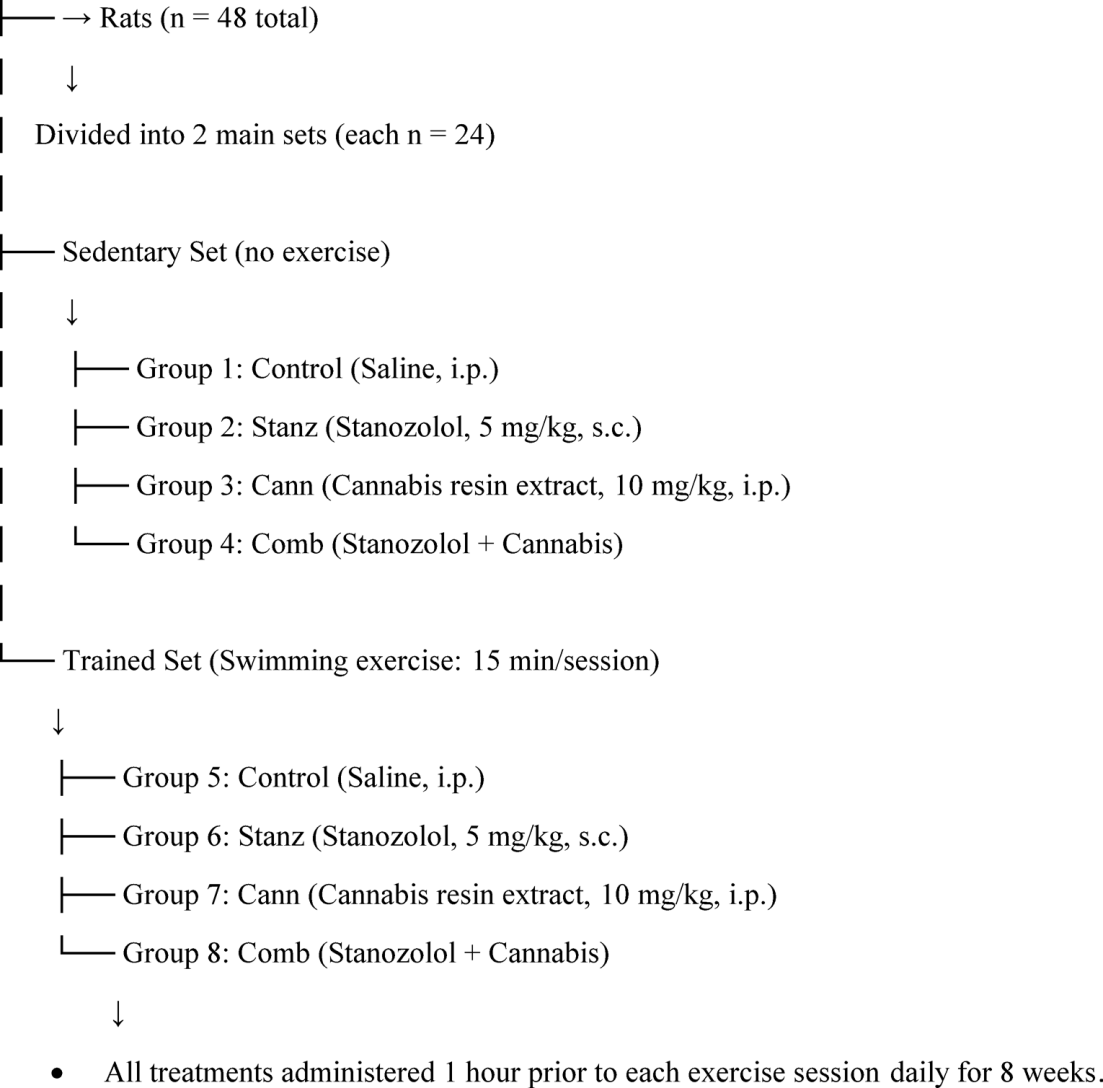

### Swimming protocol

Swimming was employed as an aerobic exercise model, following the protocol developed by Barbosa dos Santos et al. (2013) to mimic athletes’ practice^20^. The rats were housed in an 85 × 60 × 50 cm cylindrical tank with water maintained at a temperature between 30 and 31 °C. The exercise sessions lasted for an hour from 10 to 11 a.m. In order to reduce stress, the rats were gradually exposed to water in a shallow environment for a week prior to the start of the main experiment. At start, rats were allowed to exercise for 10 min, then duration and water depth were gradually increased by 5 min and 5 cm daily, till reached 20 min in 50 cm deep for five days a week.

### Sample collection and preparation

In compliance with accepted ethical standards, all rats humanely euthanized at the end of the study by intraperitoneal injection of sodium phenobarbital (40–50 mg/kg). Only when there were obvious behavioral signs of unrelieved pain—such as a hunched posture, resistance to movement, continuous belly licking, or guarding behavior—and when these symptoms persisted even after receiving analgesic medication (meloxicam, 2 mg/kg, ip) was euthanasia started. Throughout the trial period, no animal reached an early or unexpected endpoint. The rats maintained consistent health throughout the whole research, and no unexpected deaths were noted. All biological disposal after euthanasia was carried out in accordance with the stringent guidelines established by the Safety and Health Committee of the National Research Center. Blood samples were taken from the retrorbital plexus, then euthanized^21^ and the testes were excised and rinsed with saline. Testes were instantly frozen in liquid nitrogen and preserved at −80 °C till biochemical analysis. Frozen testes were homogenized in ice-cold phosphate-buffered saline (pH 7.4) to produce 20% homogenate, the homogenates were centrifuged for five minutes at 5000 xg using a cooling centrifuge (Sigma and Laborzentrifugen, 2k15, Germany).

### Biochemical analysis

#### Biochemical assays in serum

The serum levels of follicle-stimulating hormone (FSH), free testosterone, luteinizing hormone (LH) and sorbitol dehydrogenase (SDH) level were determined using commercial Enzyme-Linked Immunosorbent Assay (ELISA) kits (My BioSource, Cat No. MBS263466, MBS700807, MBS9424769, and MBS2023295, respectively), following the manufacturer’s instructions.

#### Biochemical assays in the testis

As instructed by the manufacturer, tissue N-acetyl-beta-D-glucosaminidase (NAG), myeloperoxidase (MPO), were assayed by ELISA kits (My BioSource Cat No. MBS702746 and MBS704859). Tissue malondialdehyde (MDA) was determined as thiobarbituric acid reactive substances (TBARS) measured colorimetrically at 532 nm^22^ and tissue reduced glutathione (GSH) levels determined based on Ellman’s reaction principle was read at 412 nm^23^.

### Molecular analysis

#### RNA extraction and cDNA synthesis

RNA was isolated from 100 mg of homogenized testis sample using Thermo Scientific GeneJET RNA Purification Kit, and quantified by ND-1000 Spectrophotometer (NanoDrop). cDNA was extracted from 1 mg sample of tissue-derived RNA via kit called the Revert Aid First Strand cDNA Synthesis (Thermo Scientific) and incubated in the gradient heat cycler (Bio-Rad).

#### qRT-PCR

Thermo Scientific Quantium Studio 5 Real-Time PCR System was used for qRT-PCR. SYBR Green qPCR M Thermo Scientific aster Mix was applied. A total of 20 µL of the reaction mixture consisting of 4 µL cDNA (100 ng/µL), 300 nmol/L of each set of primers for each gene, 10 µL of SYBR Green Master Mix, and and completed to 20 µL of nuclease-free water. The housekeeping gene GAPDH was used to normalize each gene expression. Table 1 lists the primer sequences for the various genes (produced by Biosearch Technologies, USA. The thermal cycling process was set as follows: an initial denaturation at 95 °C for 5 min, followed by 40 cycles comprising denaturation at 94 °C for 15 s and annealing at the appropriate melting temperature for each primer pair for 60 s, and 10 s of extension at 72 °C. Relative mRNA expression levels were quantified using the 2^−ΔΔCT^ method to calculate fold changes in gene expression.

**Table 1:** Specific primer sequences.

Gene	Forward	Reverse	Product size	Accession number
*Sirtuin 1 (Sirt1)*	TGAAGCTGTTCGTGGAGATATTTTT	CATGATGGCAAGTGGCTCAT	82	NM_001414959.1
*insulin 1 (Ins1)*	GCCCAGGCTTTTGTCAAACA	CGGGACTTGGGTGTGTAGAAG	101	NM_019129.3
*Steroid sulfatase (Sts)*	AATTTACAGGGGCGGGAAGG	TGGGGAAGACGTCCATGTTG	131	XM_063279793.1
Glyceraldehyde-3-phosphate dehydrogenase (Gapdh)	AGTGCCAGCCTCGTCTCATA	GATGGTGATGGGTTTCCCGT	248	NM_017008.4

### Histopathological examination

#### Histopathological examination

Testes were collected from all groups at end of the experiment and fixed in 10% neutral buffered formalin solution for histopathology. Tissue specimens were processed as followings, dehydrated in ascending concentration of ethanol (50,75 and 90%) 5 min per each concentration, cleared in xylene and paraffin (1:1) (thermostat 15 min; 56 °C), embedded in paraffin wax and sectioned at 4 μm thickness. Prepared slides sections were stained by hematoxylin & eosin then examined by (Olympus xc30. Tokyo. Japan)^24^.For each group, the 5 most rounded sections were randomly selected. The following values were measured using a microscope (x200 magnification) and TSView - the diameter of the seminiferous tubule was measured on the larger and smaller axis from which the mean diameter was obtained, mean seminiferous lumen diameter (MSLD) - the diameter of the seminiferous lumen was measured on the larger and smaller axis from which the mean diameter was obtained, epithelial height, tubular area, luminal area and Johnsen score^25^. Furthermore, Periodic Acid Schiff (PAS) stain was used to show degenerated germinal layers^26^.

#### Immunohistochemical examination (Caspase-3 and inducible nitric oxide synthase (iNOS)

The immunohistochemical examination of testicular tissue. Caspase-3 and inducible nitric oxide synthase (iNOS) were measured histochemical using the method described in^27^. Following deparaffinization, rehydration, and antigen retrieval using citrate buffer (pH 6), primary antibodies against Caspase 3 (YPA 1086, China) and iNOS (GB12086, China) were employed. Following the manufacturer’s instructions, a secondary horseradish peroxidase (HRP)–labeled antibody was applied (Universal PolyHRP DAB kit for mouse and rabbit; Genemed, Sakura, Torrance, CA, USA). Hematoxylin was employed as the counterstain and diaminobenzidine as the substrate. The immunohistochemical staining for Caspase-3 and iNOS was semi-quantitively assessed in ten high microscopic power fields (40X) as described by^28^ .Two main criteria, the color intensity and the percentage (%) of positively stained cells, were basically used for this assessment. For color intensity, a grading system scaled from 0 to 3 was used, in which grade 0 = no staining, graded 1 = weak staining, graded 2 = moderate staining and graded 3 = strong staining. Similarly, a grading system scaled from 0 to 3 was used for assessment the percentage (%) of positively stained cells in high power fields, in which grade 0 denotes 0%, grade 1denotes < 30%, grade 2 denotes 30%–70% and grade 2 denotes > 70%. The total immunoreactivity score (IRS) of each stained section is the sum of these two criteria.

### Statistical analysis

Statistical analyses were conducted using a two-way ANOVA to assess the main effects of exercise training and drug treatment, as well as the interaction between treatments. To evaluate differences among groups, one-way ANOVA followed by Tukey’s post-hoc test were employed to determine statistical significance. Analyses were performed using GraphPad Prism software (GraphPad Software Inc., USA). Results are presented as means with standard errors, at p-values less than 0.05 considered statistically significant.

## Results

### Serum hormonal assay and testicular enzymes evaluation

#### Serum follicle-stimulating hormone (FSH) levels

**Fig. 1:**
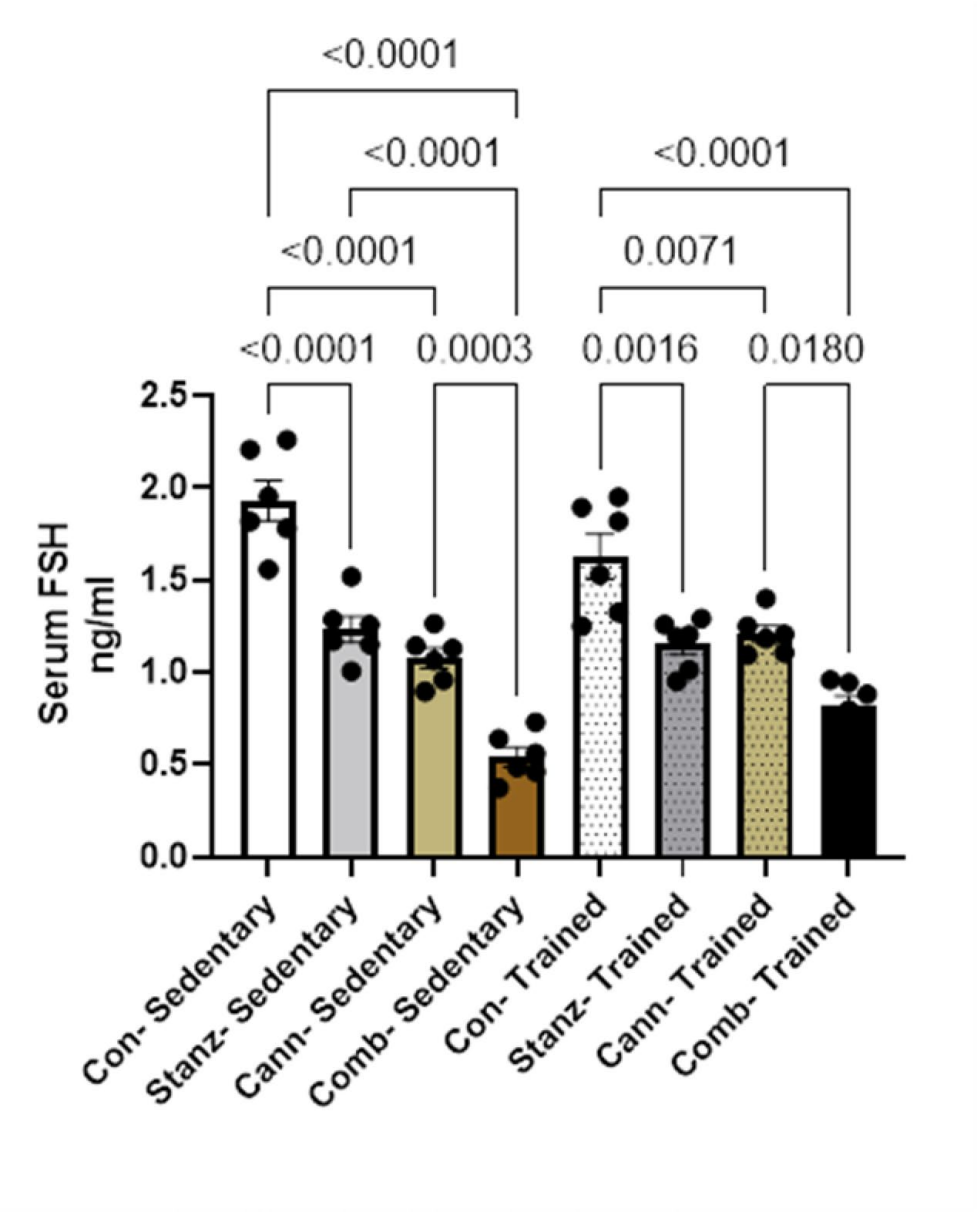
Serum FSH level across different experimental groups: Control (Con), Stanzolol (Stanz), Cannabis (Cann), and Combined treatments (Comb), with each group further exposed to Sedentary and Training conditions. Data are expressed as mean ± SEM; Significance was tested at *p *< 0.05 using one-way ANOVA followed by Tukey’s post hoc test for comparison (*n *= 6/group).

Serum FSH levels were lower (*p* < 0.0001) in the control-trained group than in the control-sedentary group (Fig. 1). Additionally, FSH levels were significantly lower in both trained groups (Stanz-Trained, Cann-Trained, and Combo-Trained) compared to their control Trained group (*p* < 0.05), After taking either cannabis and or stanozolol, the trained groups’ differences in FSH levels are less noticeable than those counter parts sedentary groups; otherwise, their combination further decreased the level in sedentary conditions.

#### Serum luteinizing hormone LH levels

**Fig. 2:**
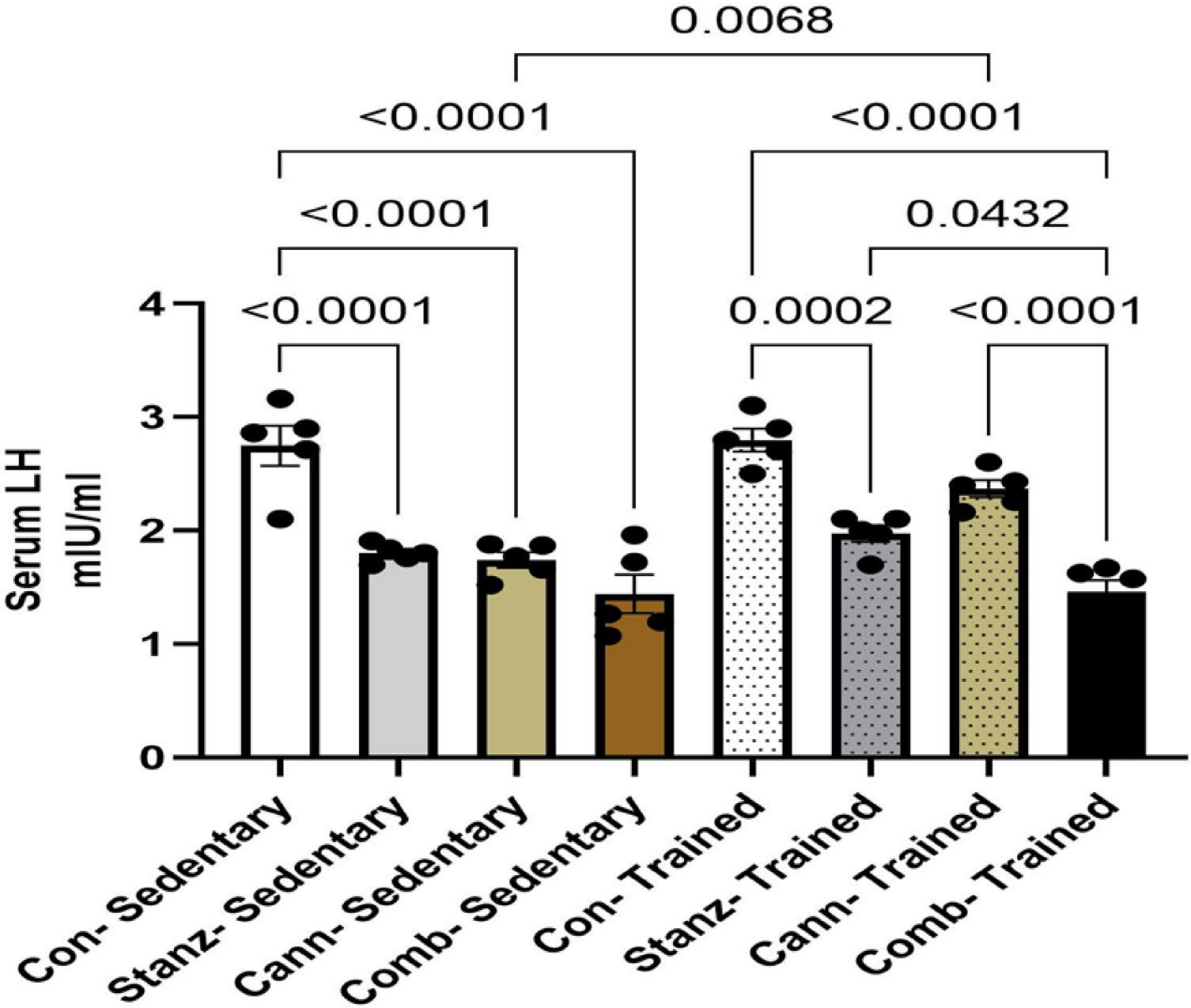
Serum (LH) level across different experimental groups: Control (Con), Stanzolol (Stanz), Cannabis (Cann), and Combined treatments (Comb), with each group further exposed to Sedentary and Training conditions. Data are expressed as mean ± SEM; Significance was tested at *p *< 0.05 using one-way ANOVA followed by Tukey’s post hoc test for comparison (*n *= 6/group).

The Control sedentary group shows the highest serum LH level (~ 3.5 IU/mL). All treated groups (Stanozolol, Cannabis, Combined) in both sedentary and trained rats show significantly lower LH levels than the control sedentary group (p-values < 0.0001). Among sedentary rats, cannabis alone and combined treatments show LH levels slightly lower than stanozolol alone. Training itself seems to reduce LH levels slightly in controls (Control Trained vs. Control Sedentary, *p* = 0.0068). The combination treatment (Comb) with training shows the lowest LH levels (~ 1.6 IU/mL), significantly lower than all others (*p* < 0.0001). (Fig. 2). This suggests that LH secretion may be regulated during training, possibly as a result of an adaptation to increased physical activity. Among the sedentary groups, the Control group had the highest levels of LH, while the cannabis and stanozolol-treated groups had noticeably lower levels. However, trained animals were affected by different treatments. Given that stanozolol and cannabis may suppress LH levels, this may imply that training could mitigate the disparities brought about by specific treatments. The results suggest that both training and some treatments have a significant effect on LH levels, with training usually having a less pronounced effect on LH level. This could be a sign of a physiological adaptation wherein increased exercise could lead to improved hormonal balance.

#### Serum free testosterone level

**Fig. 3:**
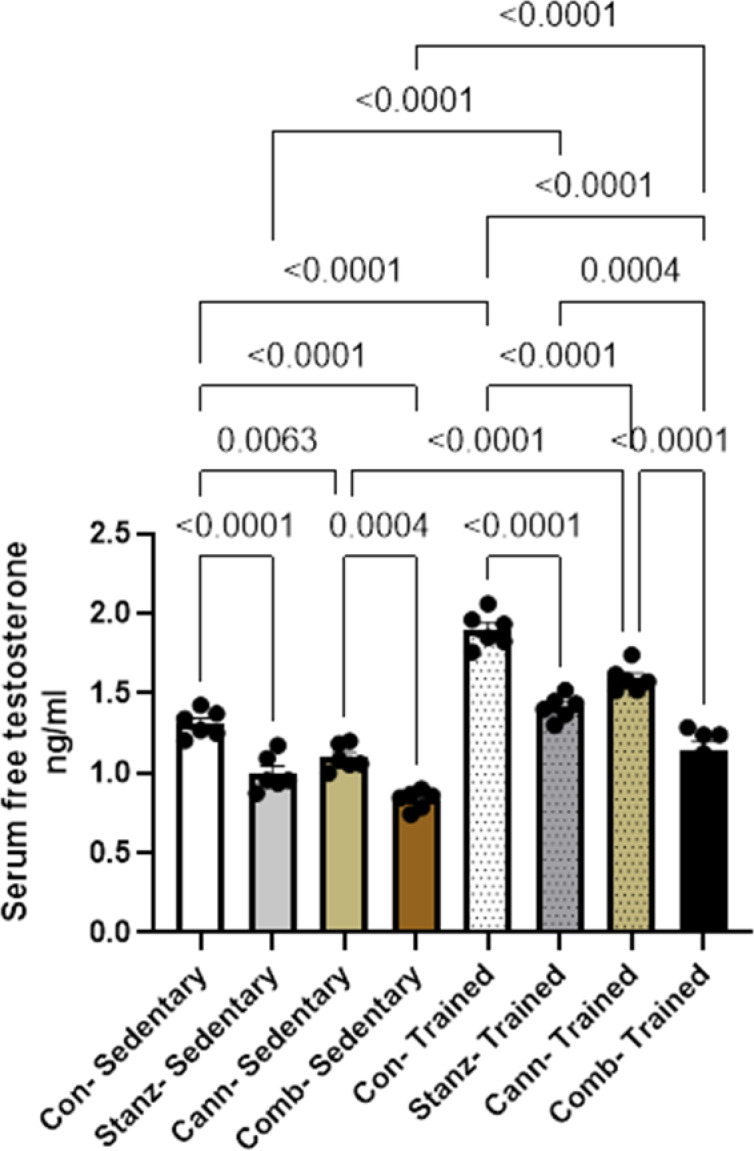
Serum Free Testosterone levels across different experimental groups: Control (Con), Stanozolol (Stanz), Cannabis (Cann), and Combined treatments (Comb), with each group further exposed to Sedentary and Training conditions. Data are expressed as mean ± SEM; Significance was tested at *p *< 0.05 using one-way ANOVA followed by Tukey’s post hoc test for comparison (*n *= 6/group).

The trained groups generally show higher testosterone levels than the sedentary groups. Among trained groups, Stanz–Trained appears to have the highest free testosterone. Comb–Sedentary has the lowest testosterone of all groups Fig. 3.

#### Serum sorbitol dehydrogenase

**Fig. 4:**
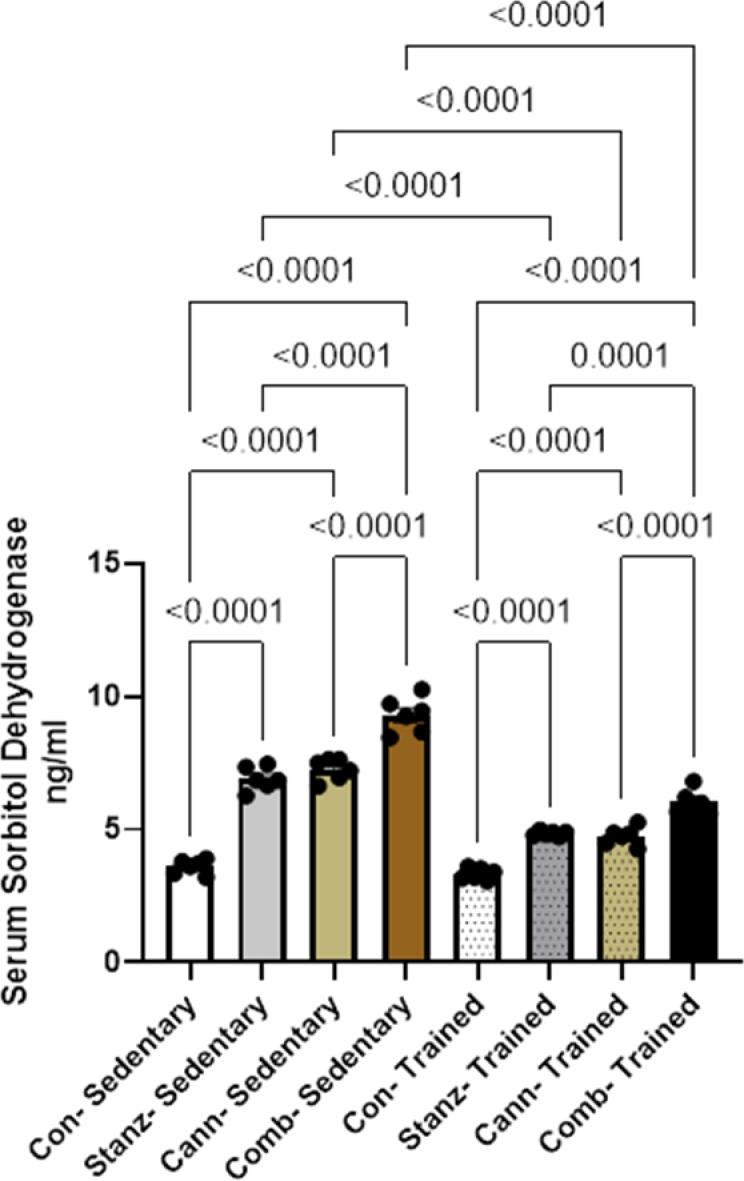
Serum Sorbitol dehydrogenase level across different experimental groups: Control (Con), Stanozolol (Stanz), Cannabis (Cann), and Combined treatments (Comb), with each group further exposed to Sedentary and Training conditions. Data are expressed as mean ± SEM; Significance was tested at *p *< 0.05 using one-way ANOVA followed by Tukey’s post hoc test for comparison (*n *= 6/group).

Serum sorbitol dehydrogenase levels were lower after training than after a sedentary condition Fig. 4. This implies that physical exercise may improve metabolic effectiveness and lessen blood sorbitol buildup. The combined group had the highest amount of sedentary behaviour among the groups. Following treatment with either cannabis or stanozolol, in training groups a noticeable metabolic response was observed.

### Measurement of oxidative stress biomarkers in testis homogenates

**Fig. 5:**
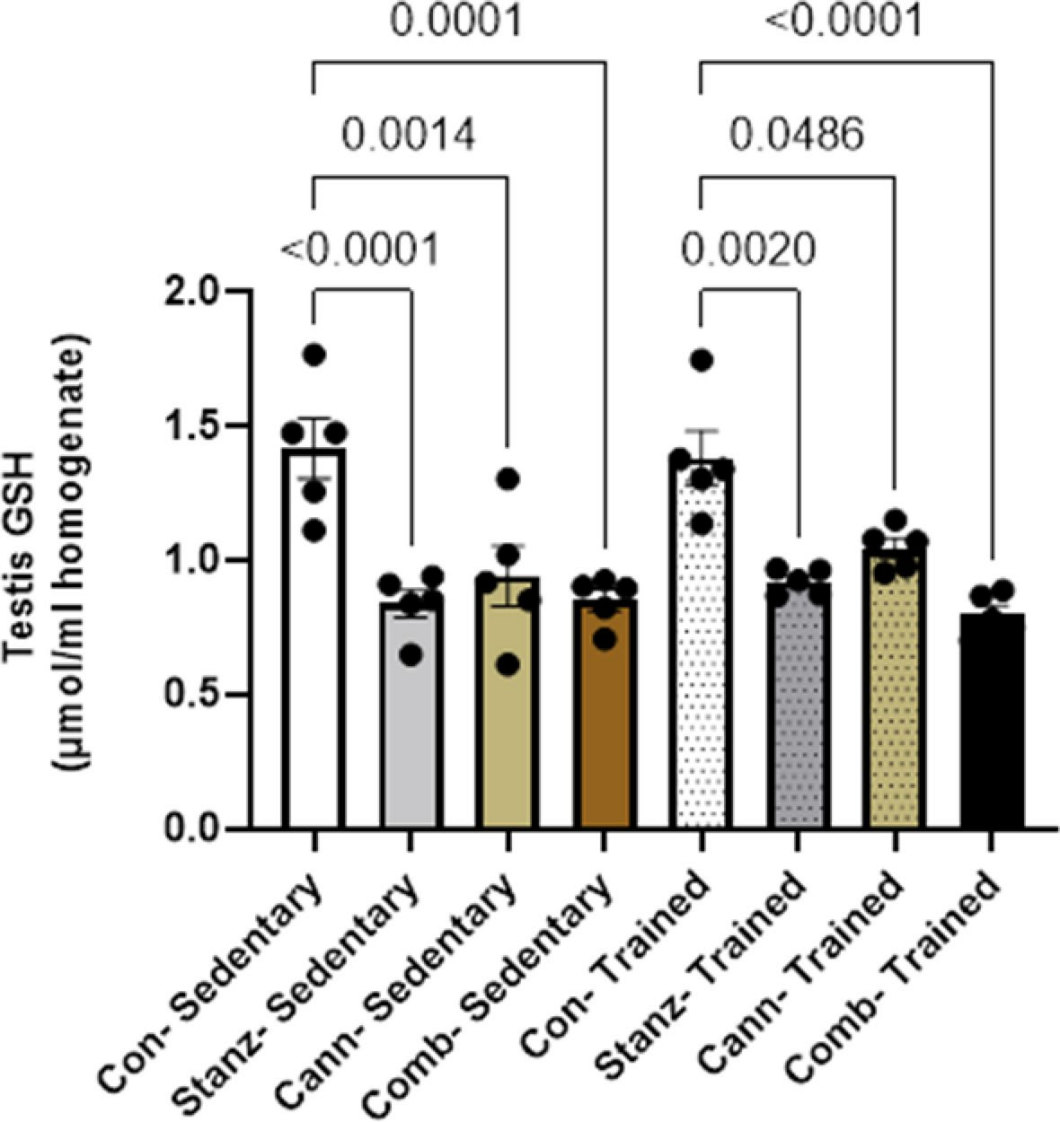
GSH level in testicular homogenate across different experimental groups: Control (Con), Stanozolol (Stanz), Cannabis (Cann), and Combined treatments (Comb), with each group further exposed to Sedentary and Training conditions. Data are expressed as mean ± SEM; Significance was tested at *p *< 0.05 using one-way ANOVA followed by Tukey’s post hoc test for comparison (*n *= 6/group)

**Fig. 6:**
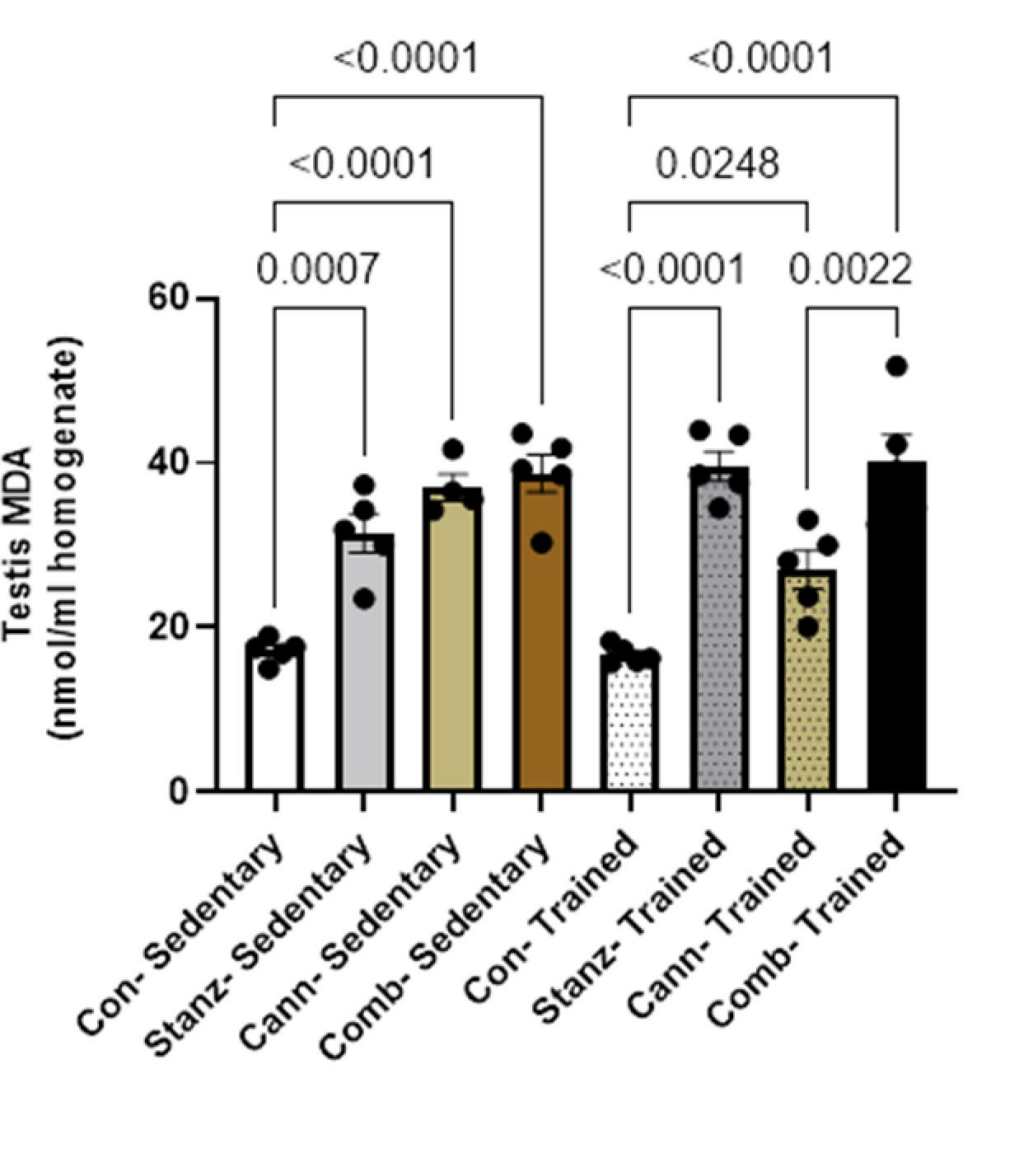
MDA in testicular homogenate across different experimental groups: Control (Con), Stanozolol (Stanz), Cannabis (Cann), and Combined treatments (Comb), with each group further exposed to Sedentary and Training conditions. Data are expressed as mean ± SEM; Significance was tested at *p *< 0.05 using one-way ANOVA followed by Tukey’s post hoc test for comparison (*n *= 6/group)

In training conditions, the groups received cannabis and or Stanozolol generally showed lower GSH and greater MDA levels when compared to control group Figs. 5 and 6. Also, the sedentary groups treated with cannabis and Stanozolol showed considerably higher MDA and significantly lower GSH levels.

### Testicular NAG (N-acetyl-β-glucosaminidase) levels

**Fig. 7:**
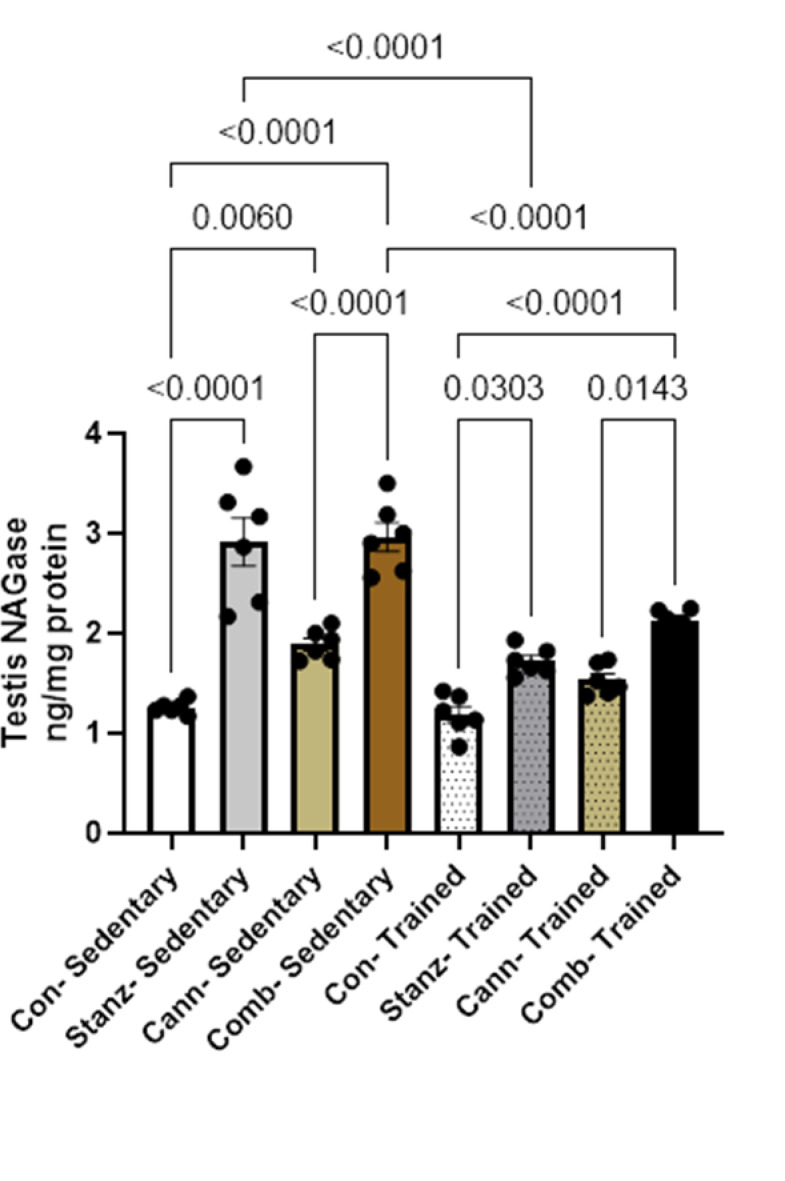
NAG levels in testis tissue across different experimental groups: Control (Con), Stanozolol (Stanz), Cannabis (Cann), and Combined treatments (Comb), with each group further exposed to Sedentary and Training conditions. Data are expressed as mean ± SEM; Significance was tested at *p *< 0.05 using one-way ANOVA followed by Tukey’s post hoc test for comparison (*n *= 6/group).

Stanozolol, cannabis, and their combined administration resulted in significant increase in NAG levels relative to the trained groups (*p* < 0.05), suggesting damaging effect of these substances on testicular lysosomal enzyme activity. Although training tended to decrease NAG levels within each treatment group, (Fig. 7). This could suggest that training lowers this enzyme’s activity, possibly as a physiological response to increased exercise. The control group had lowered NAG levels among the sedentary groups, indicating that sedentary conditions may boost enzyme activity. When compared to the control group, cannabis and stanzolol dramatically increase NAG levels, suggesting that these medications have damaging effect on testis. Training may lessen variability in NAG activity independent of the particular therapy administered, as seen by the trained groups’ less noticeable changes in NAG levels across treatments.

### Testicular myeloperoxidase MPO

**Fig. 8:**
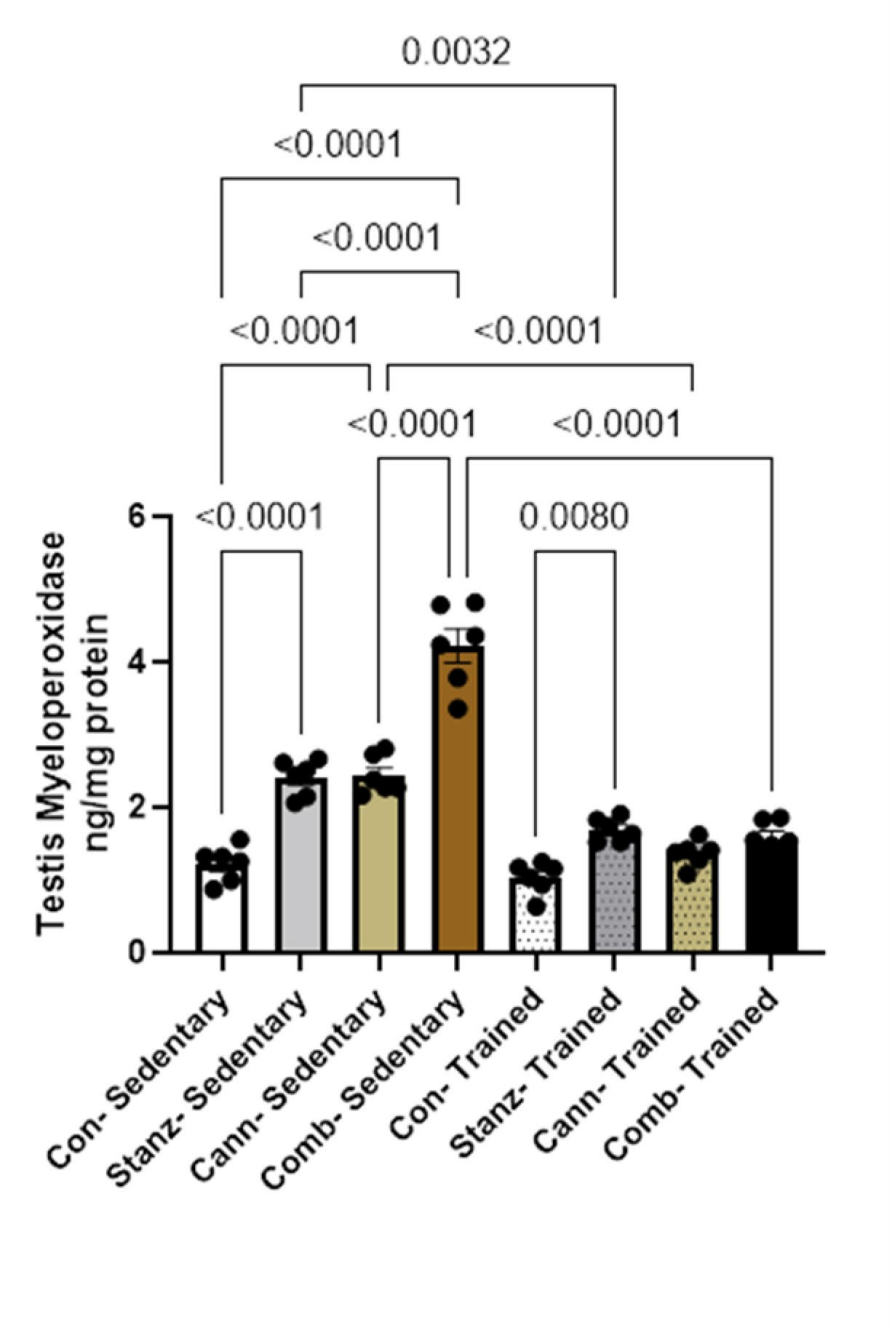
MPO levels in testis tissue across different experimental groups: Control (Con), Stanozolol (Stanz), Cannabis (Cann), and Combined treatments (Comb), with each group further exposed to Sedentary and Training conditions. Data are expressed as mean ± SEM; Significance was tested at *p *< 0.05 using one-way ANOVA followed by Tukey’s post hoc test for comparison (*n *= 6/group).

MPO levels were generally lower in the trained groups than in the sedentary groups (Fig. 8). This suggests that exercise may lower myeloperoxidase activity, which could be a sign of better testicular inflammatory response or oxidative stress control. The control group has the lowest MPO levels among the sedentary groups, indicating that a lack of exercise may be linked to higher enzyme activity. However, sedentary rats exposed to cannabis and stanozolol had noticeably increased MPO levels. In general, MPO levels were lower in the trained groups than in the sedentary groups (Fig. 8). This explains that exercise may lower myeloperoxidase activity, which may be a feature of better testicular inflammation response or oxidative stress management. The trained groups may appear visually closer, the data support the conclusion that training consistently decreases variability.

### Quantitative real-time PCR

**Fig. 9:**
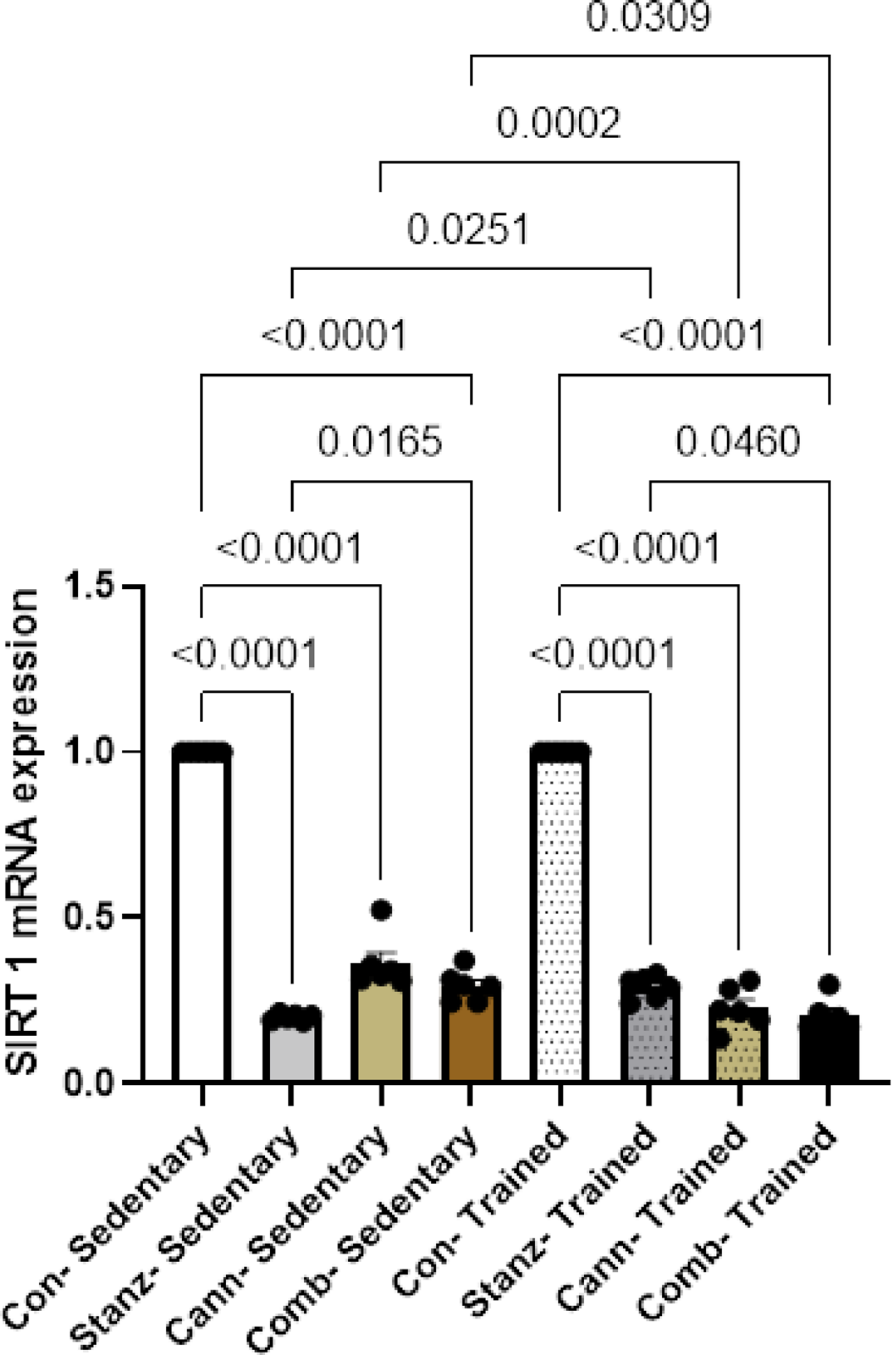
SIRT1 expression levels in testis tissue across different experimental groups: Control (Con), Stanozolol (Stanz), Cannabis (Cann), and Combined treatments (Comb), with each group further exposed to Sedentary and Training conditions. Data are expressed as mean ± SEM; Significance was tested at *p *< 0.05 using one-way ANOVA followed by Tukey’s post hoc test for comparison (*n *= 6/group).

**Fig. 10:**
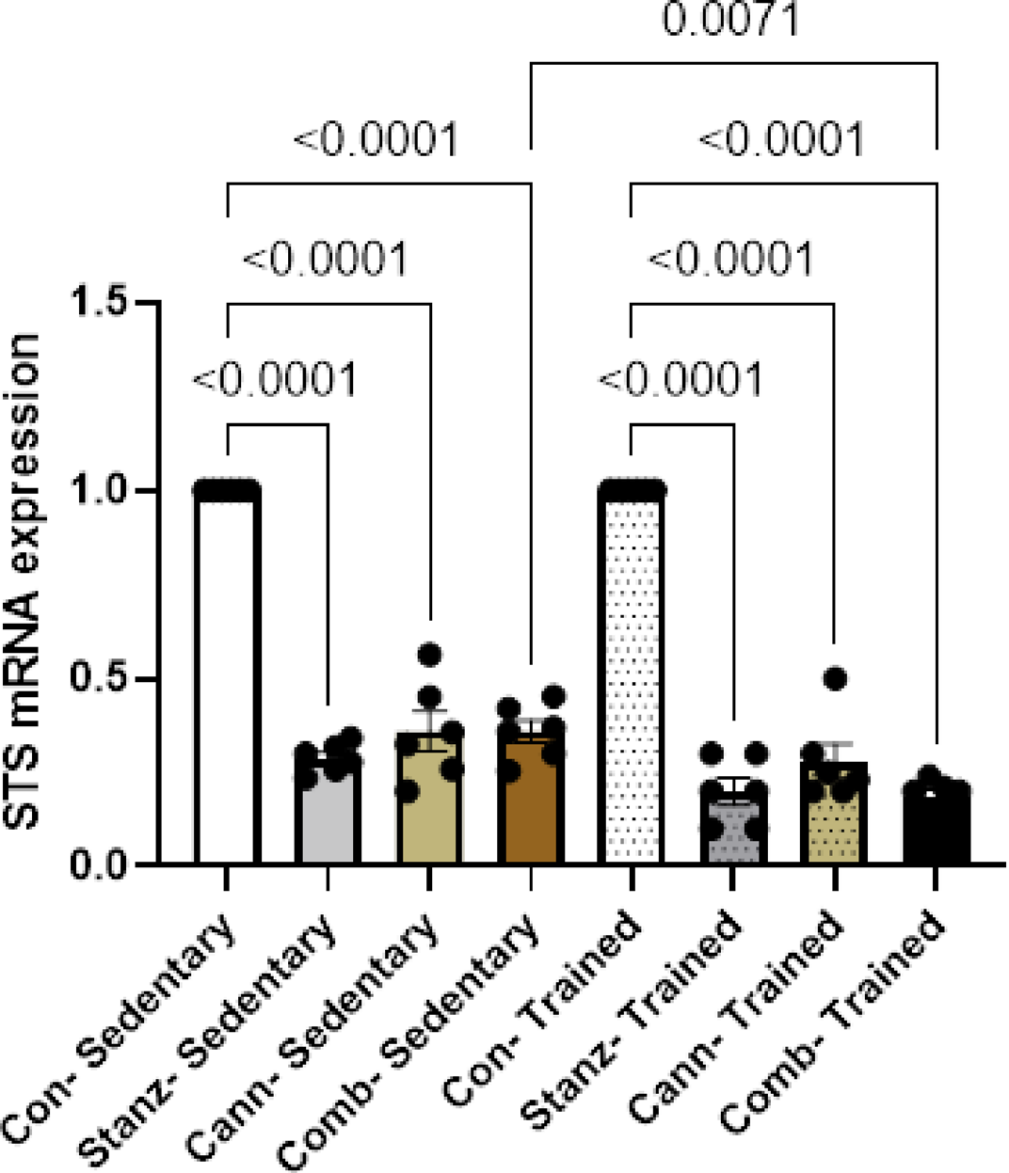
STS expression levels in testis tissue across different experimental groups: Control (Con), Stanozolol (Stanz), Cannabis (Cann), and Combined treatments (Comb), with each group further exposed to Sedentary and Training conditions. Data are expressed as mean ± SEM; Significance was tested at *p *< 0.05 using one-way ANOVA followed by Tukey’s post hoc test for comparison (*n *= 6/group).

**Fig. 11:**
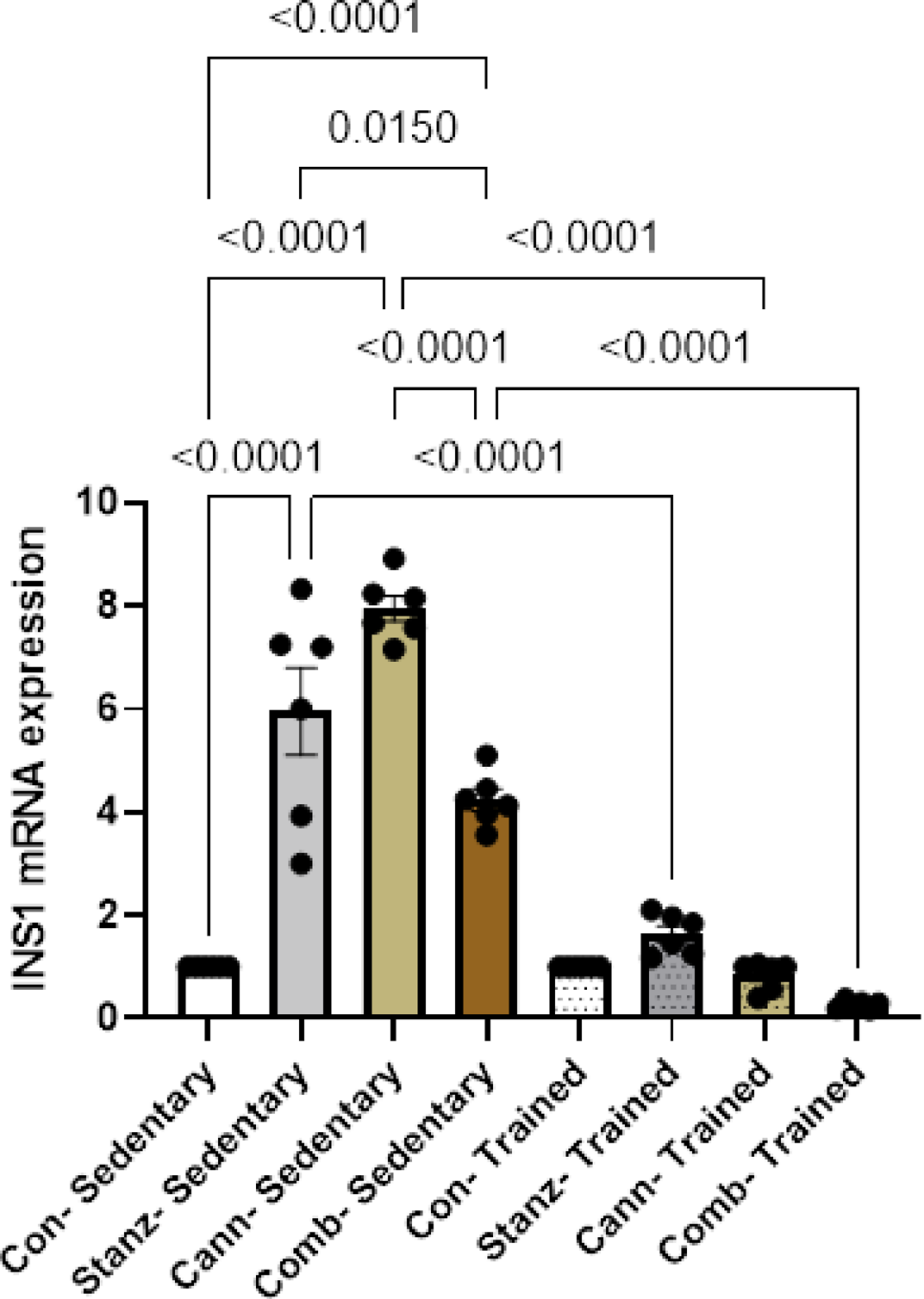
Insulin expression levels in testis tissue across different experimental groups: Control (Con), Stanozolol (Stanz), Cannabis (Cann), and Combined treatments (Comb), with each group further exposed to Sedentary and Training condition. Data are expressed as mean ± SEM; Significance was tested at *p *< 0.05 using one-way ANOVA followed by Tukey’s post hoc test for comparison (*n *= 6/group).

Administration of stanozolol and or cannabis for two months showed downregulation of both SIRT1, STS and INS1genes expressions Figs. 9, 10, 11. Training has less damaging effect on testis.

### Histopathological examination of testis

#### Haematoxylin and eosin staining (H & E)

**Fig. 12:**
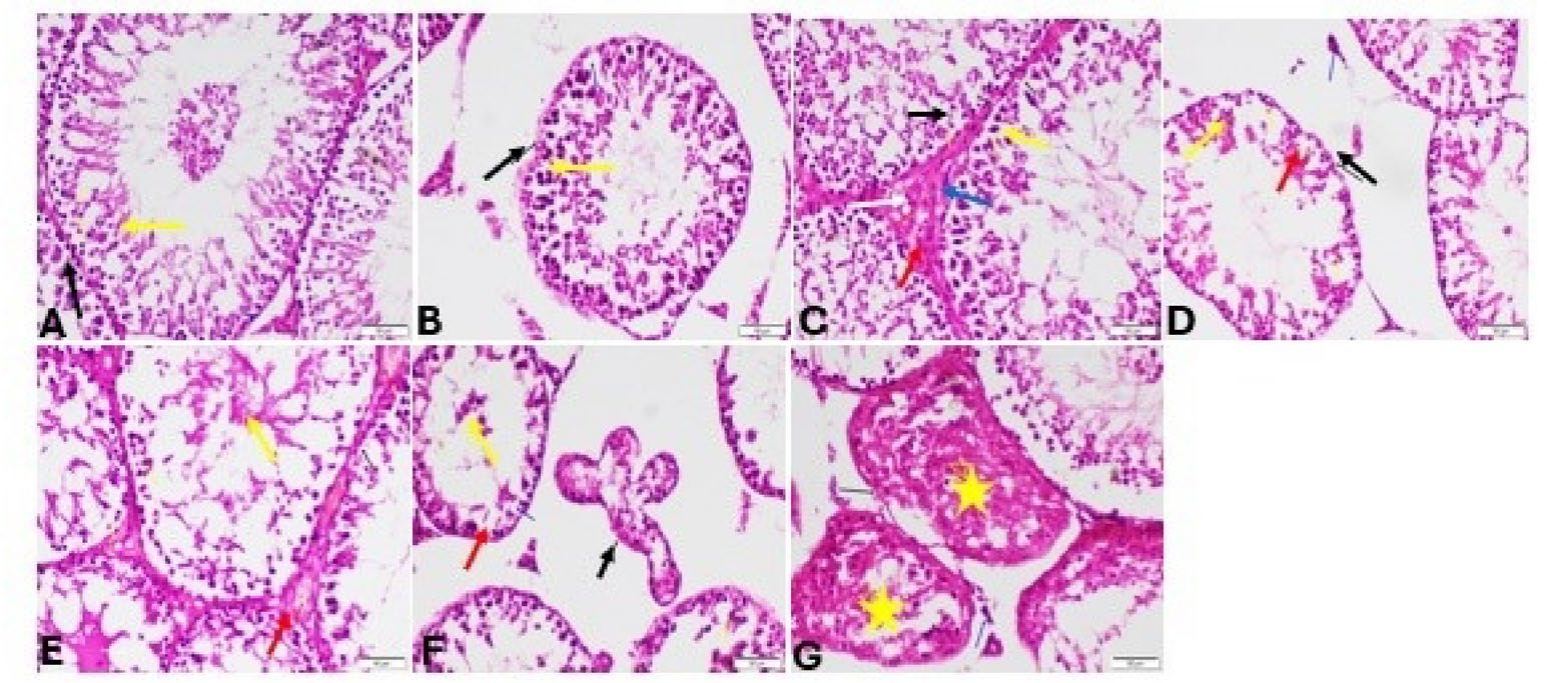
Testis sections stained by H&E for sedentary rats. (**A**): Control group showing normal histological structure of testis intact seminiferous tubule (black arrow), normal germinal layers (yellow arrow) and normal Leydig cell (blue arrow) (**B** and **C**): Stanz group (**B**) showing necrosis of germinal layers (yellow arrow) and undulating basement membrane of seminiferous tubules (balack arrow) as well as degeneration of leyding cell (blue arrow). (C) showing degeneration of germinal cells (yellow arrow), congested blood vessels (red arrow) and edema (red star). (**D** and **E**) Cann group showing necrosis of germinal layers (yellow arrow) and undulating basement membrane of seminiferous tubules (black arrow) as well as degeneration of leyding cell (blue arrow) (**E**): degeneration of germinal cells (yellow arrow), congested blood vessels (red arrow). (**F** and **G**) combined group showing evacuation of all seminiferous tubules from germinal epithelium (yellow star), undulating basement membrane (black arrow), atrophy of Leydig cells (blue arrow) and wide interstitial space (red arrow) (**G**): degeneration of all seminiferous tubules and all germinal layers (yellow stars)

**Fig. 13:**
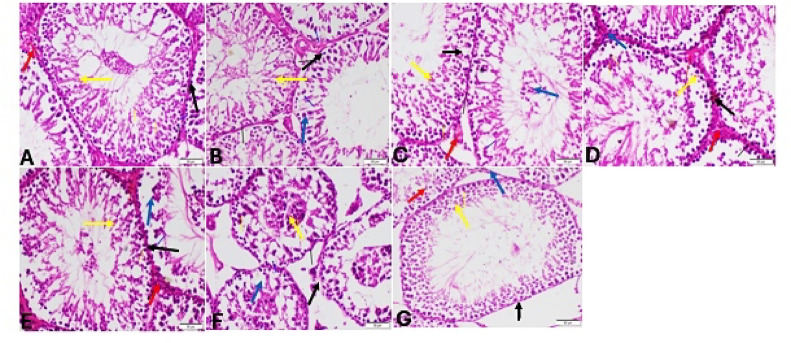
Testis sections stained by H&E, for trained rats. (**A**) Control group showing normal histological structure of testis intact seminiferous tubule (black arrow), normal germinal layers (yellow arrow) and normal Leydig cell (red arrow). (**B**, **C**), Stanz group intact seminiferous tubules (black arrow), regular arrangement of all germinal layers and healthy spermatids (yellow arrow), as well as degeneration of spermatogenic cells (blue arrow). (**C**) Showing intact seminiferous tubules (black arrow), regular arrangement of all germinal layers and healthy spermatids (yellow arrow), congested blood vessel (red arrow), degeneration of spermatogonia (blue arrow). (**D**, **E**) Cann group (**D**): Showing intact seminiferous tubules (black arrow), hyperplasia of Leydig cell (blue arrow), oedema (red arrow), degeneration of spermatogonia (yellow arrow), (**E**): Showing intact seminiferous tubules (black arrow), regular arrangement of all germinal layers and healthy spermatids (yellow arrow), oedema (red arrow), degeneration of spermatogonia (blue arrow) (**F**, **G**) combined group. (**F**) distortion of seminiferous tubules (black arrow), exfoliation into the tubular lumen (yellow arrow), vacuolation of Sertoli cells (blue arrow) (**G**): showing mild distortion of seminiferous tubules (black arrow) and organization of the seminiferous epithelium (yellow arrow), degeneration of some germ cell (red arrow) and atrophy of Leydig cells (blue arrow)

Examining the testicular tissue of sedentary groups revealed normal seminiferous tubules bordered with a profusion of Sertoli cells, many mitotically active spermatogonia, undamaged germinal layers, and normal Leydig cells in the control group of sedentary rats (Fig. 12A). Cconsiderable testicular degeneration was demonstrated in the sedentary rats administered Stanz or Cann, which was characterised by leyding cell degeneration, necrosis of germinal layers, and an undulating basement membrane of seminiferous tubules (Fig. 12B–E). Furthermore, the interstial tissue presented congested blood vessels, deposition of an extremely eosinophilic material and vacuolation of Sertoli cells (Fig. 12B–E). In contrast, combined group revealed severe histopathological changes of testicular tissues including vacuity of all seminiferous tubules from germinal epithelium, undulating basement membrane, atrophy of Leydig cells and wide interstitial space (Fig. 12F,G). Control group of training rats displayed normal histological structure of testis (Fig. 13A). In contrast, moderate improvement in training rats receiving either Stanz or Cann was shown compared to sedentary Stanz and Cann groups to include intact seminiferous tubules, regular arrangement of all germinal layers and healthy spermatids as well as mild degeneration of spermatogenic cells (Fig. 13B–E). Moreover, Cann group showed hyperplasia of interstial tissue (Fig. 13D,E). Furthermore, (Fig. 13F,G) showed slight improvement of pathological changes in training receiving combined treatment however, deforming of seminiferous tubules, disorganization of the seminiferous epithelium, exfoliation into the tubular lumen, germ cell and the tubular basement membrane were separated as well as atrophy of Leydig cells was detected. The changes in diameter of seminiferous tubules and tubular height were illustrated in Table 2.

**Table 2:** Basic morphological analyses including diameter of seminiferous tubules and tubular height.

Groups Parameters	Con sedentary	Stanz- sedentary	Cann- sedentary	Comb- sedentary	Con- trained	Stanz- trained	Cann- trained	Comb- Trained
Seminiferous lumen diameter	29.36 ± 3.140	18.45± 0.79^*^	16.2 ± 0.90*	12.34 ± 0.69*	29.48 ± 2.71	21.272 ± 1.09*	18.94 ± 0.64*	14.8 ± 0.61*
Epithelium height	6.66 ± 0.33	4.4 ± 0.14^*^	3.44 ± 0.17*	1.84 ± 0.21*	6.5 ± 0.41	5.34 ± 0.14*	4.48 ± 0.30*	3.1 ± 0.43

Data expressed as Control (Con), Stanzolol (Stanz), Cannabis (Cann), and Combined treatments (Comb), with each group further exposed to Sedentary and Training conditions. Data are expressed as mean ± SD; Significance was tested at *p* < 0.05 using one-way ANOVA followed by Tukey’s post hoc test for comparison (*n* = 5/group).

#### Periodic acid-Schiff (PAS) staining

**Fig. 14:**
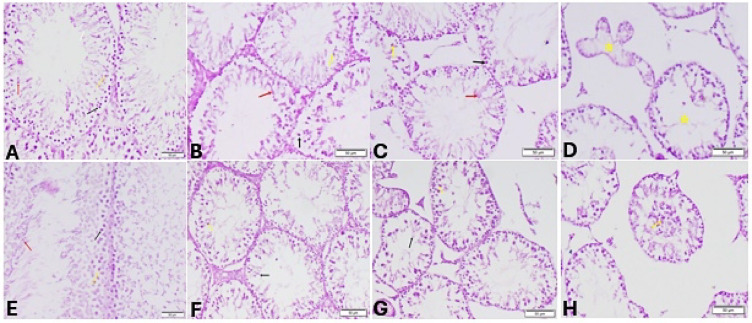
Testis sections stained by Periodic acid-Schiff (PAS) staining for sedentary (**A**–**D**) and trained (**E**–**H**) rats. Sedentary groups: (**A**) Control group (sedentary) showing normal spermatogonia (black arrow), spermatocytes (yellow arrow), round (red arrow) and elongated spermatids (white arrow), (**B**): Stanz group Showing degeneration of pachytene spermatocytes (yellow arrow) and elongated spermatids (red arrow), (**C**) Cann group showing degeneration of spermatogenic cell (black arrow) pachytene spermatocytes (yellow arrow) and elongated spermatids (red arrow). (**D**) Combined group showing absence of all germinal layers (yellow star). Trained groups: (**E**) Control group trained Showing normal spermatogonia (black arrow), spermatocytes (yellow arrow), round and elongated spermatids (red arrow). (**F**): Stanz group showing degenerated round (yellow arrow) and elongated spermatids (black arrow). (**G**) Cann group Showing degenerated spermatocyte (yellow arrow), and degenerated elongated spermatids. (**H**) Combined group Showing distorted elongated spermatids with exfoliated germinal cells (yellow arrow).

Segments of the testes from the sedentary control group stained with Periodic Acid-Schiff (PAS) displayed normal spermatogenesis with well-organized phases of germ cell development, including spermatogonia, spermatocytes, and spherical and elongated spermatids (Fig. 14A). Furthermore, sedentary Stanz and Cann groups displayed elongated spermatids and pachytene spermatocytes degenerating (Fig. 14B,C). Conversely, the sedentary combined group exhibited lack of all germinal layers and significant spermiogenesis abnormalities (Fig. 14D). On the other hand, the control group of trained rats displayed normal spermatogenesis (Fig. 14E). Stanz and Cann trained groups showed slight organized stages of germ cell development and presence of degenerated round and elongated spermatids (Fig. 14F,G). However, trained combined group experienced severe defective spermatogenesis with abnormal acrosomal caps and distorted elongated spermatids (Fig. 14H).

#### Immunehistochemical examination of testis

**Fig. 15:**
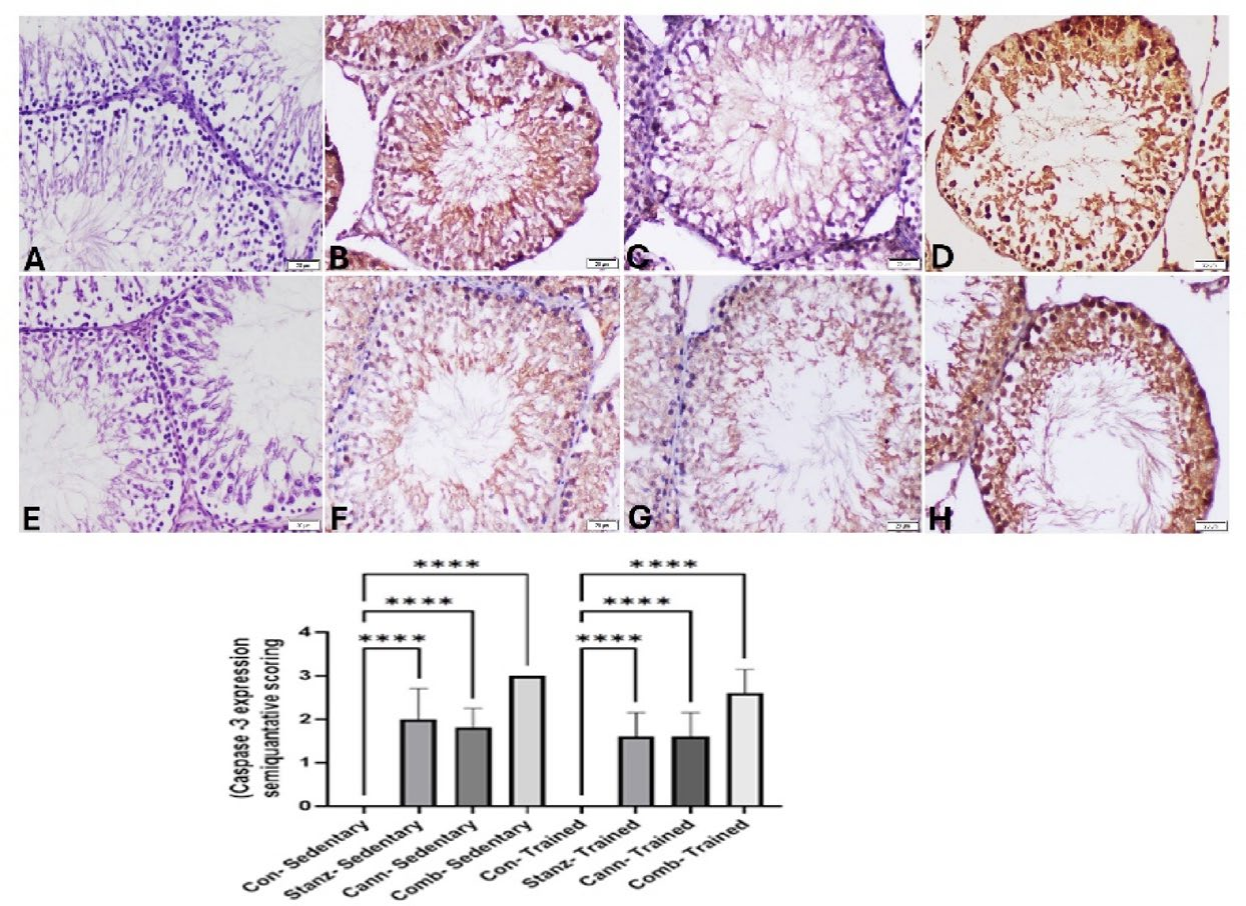
Testis sections immunostained for caspase-3 in sedentary (**A**–**D**) and trained (**E**–**H**) rats. Sedentary groups: (**A**): Control group showing no caspase-3 expression, (**B**): Stanz group demonstrating strong brown staining of germ cells, (**C**): Cann group showing positive caspase-3 expression in most of seminiferous tubules. (**D**): Combined group showing strong brown cytoplasm and nuclear staining in all germ cells. trained groups (**E**): Control rats showing no caspase-3 expression in the testes. (**F**): Stanz group with moderately positive stained cells in the seminiferous tubules. (**G**): Cann group showing moderate positively stained cells in the seminiferous tubules, (**H**): Combined group with scattered spermatogonia, and spermatocyte cells with brown nuclear staining. The bar graph represents the score of stained tissue and expressed as mean ± SEM, significance at *p *< 0.05 using one-way ANOVA followed by Tukey’s multi-comparison test (*n *= 6).

No significant Caspase-3 expression was demonstrated in the testis of the control rats of either sedentary or trained groups (Fig. 15A,E). In contrast, increased Caspase-3 expression was recorded in sedentary rats exposed to Stanz or Cann (Fig. 15B,C). Strongly stained cells were found in testis of sedentary combined group (Fig. 15D). On the other hand, after training exercises, caspase-3 expression was much lowered in the Stanz and Cann groups (Fig. 15F,G). Additionally, the trained combined group showed a slight decrease in Caspase-3 expression (Fig. 15H).

**Fig. 16:**
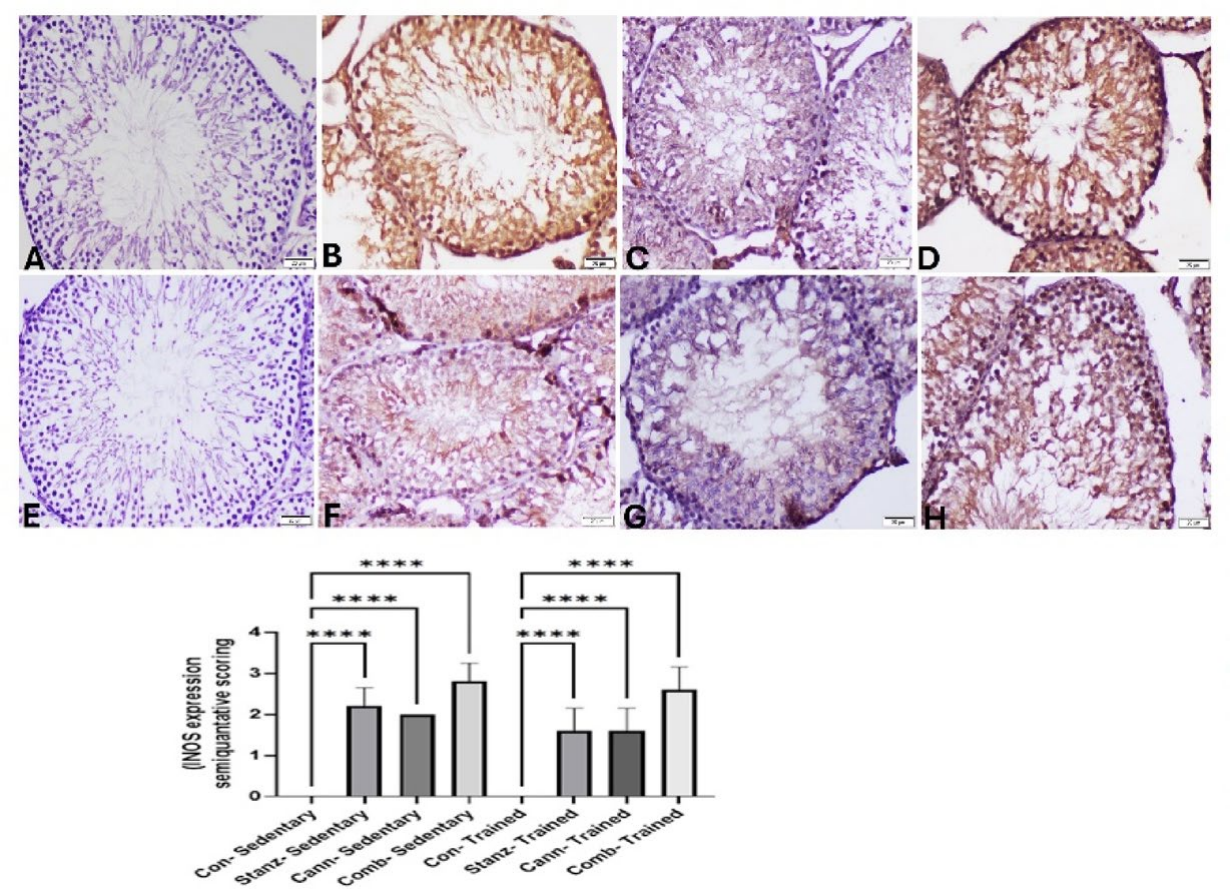
Testis sections of iNOS immunoexpression in testis sections of sedentary (**A**-**D**) and trained (**E**-**H**) rats. Sedentary groups: (**A**) Control group showing no iNOS expression, (**B**): Stanz group, Numerous strong positively stained cells in testicular tissue. (**C**): Cann group showing remarkable increase of iNOS expression of all germ cells. (**D**): Combined group Intense brown staining of spermatogonia and spermatocytes. trained groups: (**E**): Control group showed no iNOS expression, (**F**): Stanz group Moderate cytoplasmic staining of germ cell. (**G**): Cann group Scattered weakly stained spermatocytes cells, and (H): Combined group Abundant positively spermatogonia, spermatocytes, spermatids, Sertoli cells, and Leydig cells. The bar graph represents the score of stained tissue and expressed as mean ± SEM, significance at *p *< 0.05 using one-way ANOVA followed by Tukey’s multi-comparison test (*n *= 6).

Testicular iNOS immunoexpression was significantly altered by the studied treatments. The control groups either sedentary or trained rats have not expressed iNOS in testicular tissue (Fig. 16A,E). In contrast, the Stanz or Cann sedentary groups showed that the cytoplasm of testicular cells had a brown hue, which indicated increased expression of iNOS (Fig. 16B,C). iNOS positive cells with strong brown staining were found in greater number in the sedentary combined group within all germ cells (Fig. 16E). In the Stanz and Cann trained group, there was a moderate decrease of induced iNOS expression (Fig. 16F,G). However, mild amelioration of iNOS expression was recorded in the combined trained group (Fig. 16H).

## Discussion

Although adolescents often co-abuse cannabis and stanozolol in sports contexts, the long-term consequences of this combined drug use remain poorly understood. According to previous reports and anti-doping statistics, cannabis and stanozolol are among the most frequently abused substances in competitive and recreational sports. Despite their distinct pharmacological actions, the concurrent use of these drugs may exert synergistic or antagonistic effects on physiological and behavioural outcomes, which warrants further investigation^29^. furthermore, stanozolol use has been linked to multiple documented fatalities^30^. Our findings suggest a potential role of inflammation and oxidative stress and provide novel insight into the combined detrimental effects of stanozolol on testicular health, irrespective of exercise.

The proper function of the male reproductive system is largely regulated by the hypothalamus-pituitary-testicular (HPT) axis. Gonadotropin-releasing hormone (GnRH) from the hypothalamus stimulates the anterior pituitary to produce and release the gonadotropins, follicle-stimulating hormone (FSH) and luteinizing hormone (LH). Within the seminiferous tubules, these hormones drive spermatogenesis by acting on Sertoli and peritubular cells. LH also stimulates Leydig cells to produce testosterone, while FSH binds to Sertoli cell receptors to support sperm development. Testosterone, the primary male sex hormone produced by the testes, serves as a reliable indicator of testicular function^31^. Maintaining secondary sexual traits, encouraging the normal growth and development of male sex organs, and improving sperm motility, epididymal functions, and shape all depend on testosterone^32^.

The present study demonstrated a significant reduction in serum testosterone, FSH, and LH levels in groups treated with stanozolol and/or cannabis under sedentary conditions, consistent with previous reports^33^. These results are in parallel with those of Mowaad et al. (2022) The study reporting that Tramadol (TRAM) and Boldenone Undecylenate (BOLD) — alone and in combination — showed deteriorated testicular function and lowered serum levels of FSH, LH and testosterone^34^.

Our results indicate that physical training exerts a significant influence on hormone regulation. In the trained groups, FSH levels were less markedly affected by either cannabis or stanozolol alone compared to the sedentary control group; however, the combination of both substances still led to a reduction in hormonal levels. This finding may reflect a physiological adaptation, in which increased physical activity modulates hormonal balance. Previous studies have also reported that exercise can alter circulating levels of certain hormones^35^. In accordance with previous data, our results support the notion that exercise influences the reproductive hormones, where plasma levels of LH and testosterone were increased after six months of physical activity by 25% and 21%, respectively^36^.

The present study demonstrated that stanozolol, cannabis, or their combination reduced testosterone levels under both sedentary and training conditions. This notable decrease may result from inhibition of gonadotropin-releasing hormone (GnRH) secretion from the hypothalamus and subsequent suppression of LH release from the pituitary, a well-recognized effect of exogenous anabolic steroid administration^37^. The anabolic androgenic-receptor complex reduces LH and FSH secretion. Normally, the hypothalamus releases GnRH, which regulates LH. In order to regulate tissue growth and maintenance, LH combines with Leydig cell receptors to produce testosterone, which is subsequently delivered to the testis and other reproductive organs. The elevated androgen level following exogenous AAS therapy causes the pituitary gland to release LH, which in turn aids in the endogenous suppression of testosterone. The impact of exercise on reproductive hormones in the elderly was studied and showed alteration of LH and testosterone^38^.

Currently, cannabis is the most widely misused substance in the world^39^. Our study showed that cannabis had a suppressive effect on LH, FSH and testosterone levels. Payne et al.^40^ found a decrease in FSH and LH levels in marijuana regular smokers, while^41^ described lower testosterone levels in chronic marijuana users. However, Vescovi et al. observed that cannabis did not affect FSH in chronic marijuana users who received intravenous GnRH.

Oxidative stress is a key factor in the onset of testicular dysfunction, profoundly affecting testicular inflammation, spermatogenesis, sperm function, testosterone production, and overall reproductive outcomes^42,43^. Overproduction of reactive oxygen species (ROS) impairs sperm motility, viability, and the fertilizing capacity. Additionally, DNA fragmentation and subsequent increased risk of genetic abnormalities in offspring is encountered in such condition^44^. Inflammation plays a critical role in oxidative stress and its detrimental effects on sperm quality and overall reproductive function. Nitric oxide (NO), produced from L-arginine by inducible nitric oxide synthase (iNOS), helps regulate spermatogenesis at low concentrations. However, at elevated levels, NO contributes to the formation of reactive nitrogen species, which can damage testicular tissue. Unlike endothelial (eNOS) and neuronal (nNOS) isoforms, iNOS produces larger amounts of NO and is calcium-independent. Consequently, upregulation of iNOS is a key factor in the development of testicular oxidative stress and its associated reproductive impairments^45^. Previous research has shown that iNOS catalyzes the production of nitric oxide (NO), a key mediator in the induction of testicular oxidative stress. Although the present study did not directly measure NO levels, the observed overexpression of iNOS suggests a likely increase in NO production^46^.

Although the primary cause of male infertility remains unclear, elevated levels of reactive oxygen species (ROS) have been implicated in approximately 60% of cases. Due to its high lipid content and rapid oxygen consumption, the testis is particularly susceptible to lipid peroxidation and oxidative damage^34^. The substantial drop in testicular antioxidant enzyme GSH and increase in MDA concentrations observed in this investigation after any or both drugs administration may probably be a symptom of oxidative damage.

MDA levels are suggested as a biomarker of lipid peroxidation^47^. Overproduction of MDA results from an increase in free radicals and ROS, which damage cell membranes^48^. Therefore, the notable rise in testicular MDA and decrease in GSH level in rats administrated cannabis extract is a symptom of supraphysiological lipid peroxidation. On the contrary, Abdel-Salam et al.^49^, found that mice exposed to cannabis had lower MDA levels.

Stanozolol caused oxidative stress in the rat testis as well. Similarly, Tousson et al.^50^ reported that following boldenone injection, rabbits’ muscle tissues had higher MDA and lower levels of SOD and GSH. Additionally, Ibrahim and Said^51^ observed that intramuscular boldenone injection resulted in lipid peroxidation and DNA fragmentation, as well as suppression of total antioxidant capacity (TAC) and CAT activity in testis and renal tissue. Additionally, AAS use raises lipid peroxidation without increasing total antioxidants, according to Pinheiro et al.^52^. In a prior study^53^, the authors confirmed that injecting boldenone resulted in an increase in MDA and NO and a decrease in SOD and GSH in rat testicular tissues.

The testicular enzyme sorbitol dehydrogenase (SDH), which is known to be a predictive indication of testicular toxicity, was tested in this study. Testicular marker enzymes are indicators of proper gonadal cell differentiation that can show the extent of reproductive harm^54,55^. With the cofactor NAD, SDH reversibly catalyses the oxidation process of sorbitol to fructose. Additionally, this enzyme is present in the liver and kidney’s cytoplasm and mitochondria, as well as seminal vesicles, which are likewise in charge of delivering energy^56^. In comparison to the control group, our results showed a significant rise in SDH in the serum of the Stanozolol, cannabis and combined treated groups. The testes’ damaged integrity, which includes significant germinal cell disarray, degeneration, and the destruction of spermatogenic components, may be the cause of the elevated activity of the SDH enzyme, which then leak into the bloodstream.

The lysosomal peroxidase enzyme, MPO is expressed in cells highly producing reactive oxidants and involved in oxidative stress and inflammation^32^, hence its elevated level is an indicator of tissue damage^57^. MPO activity is used as a biomarker of inflammatory infiltration in tissues since it is majorly found in leukocytes, such as neutrophils. It catalyses the conversion of hydrogen peroxide to hypochlorous acid, which can activate pathways leading to cellular senescence or apoptosis. Additionally, MPO converts tyrosine to tyrosyl radical using hydrogen peroxide as an oxidizing agent, generating cytotoxic compounds that can induce oxidative damage^58^. In the current investigation, treatment with both cannabis and/or stanozolol induced MPO activity in the testis referring to severe inflammation and suggesting potential apoptotic pathways in the testicular tissue. In our investigation, the primary cause of the oxidative damage in the testes was the elevated MPO activity level. Our results are in line with a previously published study on the toxicity of anabolic steroids to various tissues^53^. Increased MPO activity in testicular tissue could be linked to neutrophil activation brought on by an excess of ROS.

On the other hand, the hydrolytic lysosomal enzyme N-Acetyl-/β-glucosaminidase (NAG) is necessary for the degradation and disposal of the cell membrane as well as other cellular structures. It breaks glycosides and amino sugar bonding and adds other sugar residues to proteins^59^. Protein glycosylation are either N-linked or O-linked. In the endoplasmic reticulum lumen, N-linked glycosylation occurs, transferring various sugar residues to the nitrogen of protein asparagine or arginine residues and are linked to spermatozoa and can be found in the proteins of epididymal fluid^60^. Cellular breakdown releases NAG into serum, while exocytosis releases it from cells due to its high molecular weight^61^. Serum NAG activity, has been found to be increased in many disorders. Damage to the proximal renal tubular cells increases the excretion of NAG in the urine^62^. NAG activity and the isozyme fraction were measured in the tissues of the urinary organ (kidney) and genital organs (prostate, testis, epididymis, and seminal vesicle)^63^. Additionally, patients with Type2 Diabetes who have normal to slightly elevated albuminuria already experience increases in NAG^64^. Rather than nephropathy, NAG is linked to vascular consequences of type 2 diabetes, such as neuropathy, retinopathy, and macrovascular disease^65^. Regarding to our study, there is high statistically significant difference between the groups, stanozolol and or cannabis compared to control group. Administration of these drugs aggravate the observed vascular alterations in testis.

Seven different sirtuins, referred to as sirtuin 1 (SIRT1)-SIRT7 in mammals, are encoded by sirtuins, also known as the Sir2 family^66^. There are multiple locations for sirtuins in somatic cells; for instance, the nucleus contains SIRT1, SIRT6, and SIRT7, while the mitochondria include SIRT4 and SIRT5. SIRT2 and SIRT3 are mostly found in the mitochondria and cytoplasm, but during the G2/M cell cycle transition and in response to stress, they go to the nucleus^67^. Cellular stress sensors called sirtuins control nuclear and mitochondrial activity to aid in cells’ ability to adjust to harsh circumstances^68^.

The sirtuin family’s most preserved member, SIRT1, is comparable to yeast^68^. Because of its deacetylase activity, which is based on the ratio of NAD+/NADH, SIRT1 is known as a nutrition sensor^69^.SIRT1 is the sirtuin family member who has drawn the greatest attention. The results of immunoblotting showed that the mouse testis has greater levels of SIRT1 expression. Additionally, SIRT1 has been shown to be present at different phases of spermatogenesis by immunohistochemistry^70^. The testis’s spermatogenic cells’ nuclei contained the SIRT1 protein^71^.

Our results are parallel with Coussens et al., who reported that Male SIRT1-deficient mice have infertility, which is typified by aberrant sperm maturation and poor spermatogenesis. It was discovered that Sirt1-deficient mice have comparatively smaller male reproductive gonads Additionally, SIRT1-deficient mice showed a significant decrease in sperm motility and numbers. Similarly, there were several aberrant sperm with rounder, smaller heads and spermatozoa with heads that had disconnected tails. The lack of germ cells or the high number of apoptotic spermatocytes and spermatids were two of the most common abnormalities^72^.

Steroid sulfatase (STS) is an enzyme responsible for steroid desulfation, with its primary substrates including cholesterol sulfate, estrone sulfate (E1S), and dehydroepiandrosterone sulfate (DHEAS)^73^. Human testis expresses STS and efflux transporters and uptake membrane carriers for sulfated steroids^74^. Our results are in accordance with, Hartmann et al.^75^ who described a decreased expression of STS in the testicular tissues associated with spermatogenic failure. In contrast, Lardone showed that increased *STS* gene expression and higher concentration of E2 in testicular tissue were linked to different types of infertility^76^. Spermatogenic cell death is a key mechanism regulating the population of testicular germ cells. Elevated production of reactive oxygen species (ROS) may accelerate apoptosis, a pathological effect associated with cannabis and stanozolol abuse. This increase in germ cell death can contribute to male infertility and further impair testicular function^77^. Oxidative stress plays a major role in the pathogenesis of testicular dysfunctions^78^.

## Conclusion

In summary the co-abuse of Stanozolol and Cannabis has deleterious effects on testicular structure and function, possibly through downregulation of SIRT1 and dysregulation of STS, with exercise training offering a mitigating effect., the data indicate that training significantly affects serum LH, testosterone, sorbitol levels, and metabolic regulation generally reducing them in trained rats compared to sedentary ones. Additionally, specific treatments also play a crucial role in modulating hormone levels, with certain treatments demonstrating a more damaging effect in sedentary conditions. These findings highlight the complex interplay between physical activity and administration of Stanozolol and or cannabis suggesting that both training and treatment modalities must be considered when evaluating different parameters to detect damaging effect on testes both biochemically, histopathological and immunohistochemical changes in male albino rats.

### Limitations of the study

- Recovery effects post-drug withdrawal was possibly not studied.
- While biochemical and histological changes were assessed, actual fertility parameters (e.g., sperm count, motility, mating success) may not have been evaluated. We will do this in further studies to complete our work.

## Data Availability

Data are available upon request. please contact the corresponding author if any data are requestedbody27_9_80@yahoo.com.
